# Vasoplegic syndrome in cardiac surgery: bridging therapeutic gaps with best practices and future research

**DOI:** 10.1093/icvts/ivaf075

**Published:** 2025-04-01

**Authors:** Dragana Unic-Stojanovic, Andreas Koster, Gabor Erdoes, Milan Milojevic

**Affiliations:** Faculty of Medicine, University of Belgrade, Belgrade, Serbia; Department of Anaesthesiology and Critical Care, Cardiovascular Institute Dedinje, Belgrade, Serbia; Clinic for Anaesthesiology and Interdisciplinary Intensive Care Medicine, Sana Heart Centre Cottbus, Germany and Ruhr University Bochum, Germany; Department of Anesthesiology and Pain Medicine, Inselspital, University Hospital Bern, University of Bern, Bern, Switzerland; Department of Cardiac Surgery and Cardiovascular Research, Dedinje Cardiovascular Institute Belgrade, Serbia

**Keywords:** vasoplegic syndrome, vasoplegia, cardiac surgery, cardiopulmonary bypass, methylene blue, evidence-based medicine

Vasoplegic syndrome (VS) is a common and potentially life-threatening complication of cardiac surgery with cardiopulmonary bypass (CPB). It presents a significant haemodynamic challenge that is often difficult to manage and can appear either intraoperatively, upon CPB initiation, or in the early postoperative period. Until recently, VS had been described under various terms, including low vascular resistance syndrome, catecholamine-resistant vasoplegia and post-cardiotomy VS. Moreover, it is also considered part of a broader spectrum of inflammatory responses, such as post-perfusion syndrome, vasoplegic shock or postoperative vasoplegia [1]. This inconsistent terminology and the overlap between definitions have made it challenging to accurately determine its true incidence, establish a standardized diagnostic and therapeutic framework and understand its association with adverse outcomes. Furthermore, the lack of a universally accepted definition hinders the identification of key patient and procedural risk factors that could help reduce VS-associated morbidity and mortality.

In this context, Zhu *et al.* [2] present a Best Evidence Topic review summarizing data on the use of methylene blue in patients with post-cardiac surgery VS. The review offers a valuable opportunity to assess its role in VS management and evaluate whether its clinical use is supported by robust evidence.

From a clinical standpoint, VS most often presents as a form of distributive shock within the first 24 h following cardiac surgery. Typically, affected patients develop persistently low blood pressure that does not respond to fluid therapy, together with markedly reduced systemic vascular resistance (SVR) and a preserved or even elevated cardiac output. While various definitions exist, they generally agree on core diagnostic criteria: a mean arterial pressure (MAP) <65 mmHg along with a cardiac index >2.2 l/min/m^2^ and an SVR <800 dynes·s/cm^5^. Some definitions also emphasize a poor response to vasopressors or the need for non-catecholamine agents. Elevated lactate levels and the absence of infection are also frequently considered diagnostic clues [1, 3, 4]. The term *low MAP* frequently appears in the literature, though target MAP ranges can vary significantly. It is important, however, to distinguish this persistent vasoplegia from the more common, transient hypotension seen at the initiation of CPB. Transient MAP drops often result from haemodilution or cardioplegia administration and usually respond well to vasopressor adjustments. In contrast, persistent vasoplegia arises from more complex pathophysiological mechanisms and requires greater vigilance. Notably, patients who experience a significant MAP decline immediately upon CPB initiation are at risk of developing prolonged post-CPB VS that resists conventional vasopressor therapy [5]. Reported incidence rates of VS vary from 5 to 45%, reflecting differences in patient populations, clinical definitions and the presence of risk factors [1]. When inadequately treated, VS is associated with multi-organ dysfunction, extended ICU stay and increased morbidity and mortality. Identified independent predictors of VS include advanced age, anaemia, renal impairment and sepsis; the use of angiotensin-converting enzyme inhibitors, beta-blockers and loop diuretics; and surgical factors like prolonged CPB and aortic cross-clamp times, redo procedures or complex cardiac surgery [5–7].

Although the exact pathophysiology of VS remains incompletely understood, current evidence points to the overproduction of nitric oxide (NO) and cyclic guanosine monophosphate (cGMP) as central drivers. This cascade is thought to begin with inducible nitric oxide synthase (iNOS) activation, leading to excessive NO production. The resulting upregulation of cGMP triggers, widespread, uncontrolled vasodilation, impairs vascular reactivity and reduces responsiveness to catecholamines, all of which combine to undermine the effectiveness of standard vasopressor therapy [3]. Emerging evidence suggests that additional mediators, such as prostacyclins, endotoxins, adrenomedullin, oxidative stress and endothelial dysfunction, also contribute to the impaired vascular tone and catecholamine resistance seen in VS. These factors may play a role in perpetuating the refractory hypotension observed in affected patients.

Although several studies have identified independent predictors of VS, several scoring systems are still under investigation to enhance early identification and guide preventive strategies [5–7]. While no single model has been universally validated, risk assessment algorithms incorporating patient comorbidities, preoperative medications and anticipated surgical complexity may help identify individuals at high risk for developing VS and guide perioperative management strategies. Conventional (first-line) management of VS typically involves high-dose vasopressors. However, this intervention can lead to vasopressor resistance and cause adverse effects, including arrhythmia, myocardial ischaemia and increased afterload—potentially impairing end-organ perfusion. Given these limitations, alternative treatment strategies have been the focus of growing interest. Several non-catecholamine agents have emerged as promising adjuncts. Today, many of these agents are used off-label in various clinical scenarios, highlighting the absence of standardized institutional protocols tailored to specific settings (Table 1). However, this also exposes patients to numerous adverse events that are often not attributed to non-catecholamine agents (Table 2). A recent meta-analysis by Kotani *et al.* evaluated non-adrenergic vasopressors for VS. Among the agents studied, methylene blue was the only drug to significantly reduce mortality risk (Risk Ratio [RR] 0.12, 95% confidence interval (CI) 0.02–0.95), compared to vasopressin (RR 0.89, 95% CI 0.65–1.22) and angiotensin II (RR 0.98, 95% CI 0.15–6.38) [8].

**Table 1:** Medications commonly used for the management of vasoplegic syndrome (including off-label applications)

Drug category	Drug	Dosage	Mechanism of action
Catecholamine			
	Norepinephrine	0.01–1.0 μg/kg/min	α1-adrenergic receptor agonist, vasoconstriction
	Epinephrine	0.01–0.5 μg/kg/min	α1, β1-adrenergic receptor agonist, increases inotropy
	Phenylephrine	0.1–5 μg/kg/min	α1-adrenergic receptor agonist
	Dopamine	1–20 μg/kg/min	Dose-dependent: β1 agonist at low dose, α1 agonist at high dose
Non-catecholamine			
	Methylene blue	1–2 mg/kg bolus over 15–30 min, infusion 0.25–2 mg/kg/h	Inhibits NO synthesis, blocks guanylate cyclase
	Hydroxocobalamin	5 g IV over 15 min, max 10 g	Scavenges NO inhibits iNOS and hydrogen sulphide
	Vasopressin	0.01–0.1 U/min	V1 receptor agonist, restores vasomotor tone
	Angiotensin II	2–40 ng/kg/min	AT1 receptor agonist stimulates aldosterone release
	Terlipressin	1.3 μg/kg/h or 1 mg bolus	Selective vasopressin V1a receptor activator
	Ascorbic Acid	1.5 g every 6 h, max. 6 g/day	Enhances catecholamine synthesis, antioxidant
	Thiamine	100–200 mg IV every 6–12 h	Cofactor for lactate metabolism
	Hydrocortisone	50 mg IV every 6 h or 200 mg/day	Restores adrenal response, reduces inflammation
	Calcium chloride	Bolus: 1–2 g, Infusion: 20–50 mg/kg/h	Enhances vascular smooth muscle contraction
	Sodium bicarbonate	1–2 mEq/kg IV push	Buffer’s acidosis increases the catecholamine response

AT: angiotensin; IV: intravenous; NO: nitric oxide; iNOS: inducible nitric oxide synthase.

**Table 2:** Clinical adverse effects associated with non-catecholamine pharmacologic agents

Drug	Type A^a^	Type B^b^	Type C^c^	Type D^d^	Type E^e^	Type F^f^	Interference with monitoring
Vasopressin	Negative inotropy, organ ischaemia, hyponatraemia, rare Thrombocytopaenia	Hypersensitivity			Diabetes insipidus		
Methylene blue	Vasoconstriction, haemolytic anaemia (G6PD deficiency), Methaemoglobinaemia (high-doses), serotonin syndrome (with serotonergic drugs), blue-green discolouration (skin, urine, sclera)	Anaphylaxis, Cutaneous effects (rash, urticaria)					Spuriously low SpO_2_ (pulse oximetry), interference with co-oximetry and blood as analysers.
Hydroxocobalamin	Severe hypertension, acute renal failure, oxalate nephropathy, cobalt toxicity.	Cutaneous effects (rash, redness), chromaturia (red-orange urine)	Hypokalaemia (during cyanide detoxification process)				False positive haemodialysis blood leak detector alarm, false elevations in bilirubin, creatinine, carboxyhaemoglobin, SpO_2_ readings.
Angiotensin II	Hypertension, haemorrhagic complications (gastrointestinal bleeding), cardiac complications (arrhythmias, myocardial ischaemia)	Thromboembolism (e.g., deep vein thrombosis, pulmonary embolism; ATHOS-3 trial noted increased risk)					
Ascorbic acid	Oxalate nephropathy (high doses, renal dysfunction)	Haemolytic anaemia (G6PD deficiency)					Spuriously elevated blood glucose readings with some point-of-care glucose meters.

Reproduced from Hwang *et al*. [11] with permission from Elsevier.

Classification of drug adverse effects according to WHO Collaborating Centre for International Drug Monitoring:

a Related to dosage.

b Not related to dosage.

c Related to dosage and duration of administration.

d Related to the duration of administration.

e Related to discontinuation of medication.

f Related to failure of therapy.

ATHOS-3 trial: angiotensin II for the treatment of high-output shock 3; G6PD deficiency: glucose-6-phosphate dehydrogenase deficiency; SpO_2_: oxygen saturation.

Although the current body of evidence is limited by small sample sizes, they highlight methylene blue as perhaps the most promising treatment option for VS in cardiac surgical settings. The reason for methylene blue’s potential effectiveness lies in its unique mechanism of action. Methylene blue inhibits both iNOS and guanylate cyclase, directly countering NO-cGMP-mediated vasodilation—a key driver of refractory VS. Through this pathway, methylene blue may help restore vascular tone, reduce vasopressor requirements and stabilize haemodynamics. The effects of methylene blue can be summarized as targeting three key mechanisms: (i) ‘*inhibition of iNOS*’ (prevents excess NO production by blocking iNOS activity, which is upregulated during systemic inflammation, such as in post-CPB vasoplegia); (ii) ‘*inhibition of soluble GC*’ (reduces downstream cGMP accumulation, limiting smooth muscle relaxation and preventing excessive vasodilation); and (iii) ‘*restoration of catecholamine sensitivity*’ (improves vascular responsiveness to both endogenous and exogenous catecholamine, enhancing the effectiveness of standard vasopressors). Potential complications of methylene blue use include rebound hypotension after drug clearance, haemolysis and renal dysfunction in glucose-6-phosphate dehydrogenase deficiency. Emerging therapies such as hydroxocobalamin (vitamin B12a), which acts as a NO scavenger, have shown promise in treating vasoplegia, particularly when methylene blue is contraindicated. Adjunctive agents like ascorbic acid (high-dose vitamin C) and corticosteroids have also been investigated for their potential vascular-stabilizing and anti-inflammatory effects.

## REVIEW OF EVIDENCE

A Best Evidence Topic review evaluated the safety and efficacy of methylene blue in treating VS after cardiac surgery, drawing data from randomized controlled trials (RCTs) and observational studies. Overall, the findings suggest that methylene blue is associated with significant haemodynamic improvements, reflected by increased MAP and SVR, along with reduced vasopressor requirements. While some studies also reported improved survival and better organ function, the results were inconsistent. Notably, the available RCTs were underpowered, and the observational studies applied varying definitions of VS. Further complicating interpretation, the timing of methylene blue administration differed widely across studies: some investigators administered methylene blue prophylactically in high-risk patients, such as those undergoing mechanical circulatory support implantation, while others reserved it as rescue therapy for cases unresponsive to conventional vasopressors.

Despite the methodological limitations of existing studies, the overall body of evidence supports both the efficacy and safety of methylene blue in VS. Reflecting this, the 2024 EACTS/EACTAIC/EBCP Guidelines on Cardiopulmonary Bypass in Adults have assigned methylene blue a Class IIa, Level B recommendation [9]. This endorsement aligns with current data suggesting that methylene blue can help restore haemodynamic stability in patients with refractory VS. However, caution is warranted regarding potential drug interactions. The concomitant use of methylene blue with serotonergic drugs carries a risk of severe serotonin syndrome, a concern highlighted by the Food and Drug Administration and European Medicines Agency.

## CHALLENGES AND FUTURE DIRECTIONS

The optimal timing and dosing regimen for methylene blue remains undetermined; however, recent studies suggest that early administration may confer benefits, including a reduction in ICU stay and mitigation of end-organ damage [10]. Key questions include the ideal bodyweight-based dose, infusion rate, and duration of administration. This uncertainty is further complicated by the fact that the pharmacokinetics of methylene blue in cardiac surgical patients have not been well characterized. Beyond dosing, clarifying the long-term safety profile is crucial. While available evidence suggests that methylene blue is generally safe, concerns persist regarding potential complications such as methaemoglobinaemia, serotonin toxicity and increased pulmonary vascular resistance. These risks may be especially relevant in patients undergoing complex cardiac procedures, underscoring the need for further investigation. Above all, large-scale RCTs are essential to establish whether methylene blue can deliver clinically meaningful improvements in patient outcomes, including the reduction of major adverse events.

## CONCLUSION

Methylene blue is an effective yet underutilized therapy for vasoplegic syndrome in cardiac surgery. Its impact on major clinical outcomes remains uncertain, primarily due to the scarcity of high-quality data. Nonetheless, the 2024 EACTS/EACTAIC/EBCP Guidelines on Cardiopulmonary Bypass in Adults have assigned methylene blue a Class IIa, Level B recommendation, supporting its early consideration in patients with refractory vasoplegic syndrome. Methylene blue should be administered without delay when indicated. Research efforts should prioritize refining optimal dosing regimens, further evaluating the long-term safety profile, and working towards a standardized definition of vasoplegic syndrome. Until more robust evidence becomes available, clinicians should be encouraged to integrate methylene blue into the perioperative management of vasoplegic syndrome.

## Data Availability

No data were produced for the content of this paper.

## References

[1] Datt V , Wadhhwa R, Sharma V, Virmani S, Minhas HS, Malik S. Vasoplegic syndrome after cardiovascular surgery: a review of pathophysiology and outcome-oriented therapeutic management. J Card Surg 2021;36:3749–60. 34251716 10.1111/jocs.15805

[2] Zhu MZL , Scullion J, Stroebel A, He C. Does methylene blue improve outcomes in patients with post-cardiac surgery vasoplegic syndrome? Interdiscip Cardiovasc Thorac Surg. 10.1093/icvts/ivae221 PMC11739618 39731744

[3] Ltaief Z , Ben-Hamouda N, Rancati V et al Vasoplegic Syndrome after Cardiopulmonary Bypass in Cardiovascular Surgery: Pathophysiology and Management in Critical Care. JCM 2022;11:6407. doi: 10.3390/jcm11216407. 36362635 PMC9658078

[4] Busse LW , Barker N, Petersen C. Vasoplegic syndrome following cardiothoracic surgery-review of pathophysiology and update of treatment options. Crit Care 2020;24:36. 32019600 10.1186/s13054-020-2743-8 PMC7001322

[5] Levin MA , Lin HM, Castillo JG, Adams DH, Reich DL, Fischer GW. Early on-cardiopulmonary bypass hypotension and other factors associated with vasoplegic syndrome. Circulation 2009;120:1664–71. 19822810 10.1161/CIRCULATIONAHA.108.814533

[6] Dayan V , Cal R, Giangrossi F. Risk factors for vasoplegia after cardiac surgery: a meta-analysis. Interact Cardiovasc Thorac Surg 2019;28:838–44. 30649312 10.1093/icvts/ivy352

[7] Jeppsson A , Rocca B, Hansson EC et al; EACTS Scientific Document Group. 2024 EACTS Guidelines on perioperative medication in adult cardiac surgery. Eur J Cardiothorac Surg 2024;67:ezae355. doi: 10.1093/ejcts/ezae355. 39385505

[8] Kotani Y , Belletti A, D'Amico F et al Non-adrenergic vasopressors for vasodilatory shock or perioperative vasoplegia: a meta-analysis of randomized controlled trials. Crit Care 2024;28:439. 39736782 10.1186/s13054-024-05212-7 PMC11686893

[9] Wahba A , Kunst G, De Somer F et al; EACTS/EACTAIC/EBCP Scientific Document Group. 2024 EACTS/EACTAIC/EBCP Guidelines on cardiopulmonary bypass in adult cardiac surgery. Eur J Cardiothorac Surg 2025;67:ezae354. doi: 10.1093/ejcts/ezae354. 39949326 PMC11826095

[10] Elbayomi M , Dewald O, Pathare P et al The mystery of methylene blue and its role in managing post-cardiac surgery vasoplegic shock. Ann Med 2025;57:2460770. 39903510 10.1080/07853890.2025.2460770 PMC11795744

[11] Hwang KY , Phoon PHY, Hwang NC. Adverse clinical effects associated with non-catecholamine pharmacologic agents for treatment of vasoplegic syndrome in adult cardiac surgery. J Cardiothorac Vasc Anesth 2024;38:802–19. 38218651 10.1053/j.jvca.2023.12.016

